# Ornithine Decarboxylase Activity Is Required for Prostatic Budding in the Developing Mouse Prostate

**DOI:** 10.1371/journal.pone.0139522

**Published:** 2015-10-01

**Authors:** Melissa Gamat, Rita L. Malinowski, Linnea J. Parkhurst, Laura M. Steinke, Paul C. Marker

**Affiliations:** School of Pharmacy, University of Wisconsin-Madison, 777 Highland Avenue, Madison, WI, United States of America; Northern Institute for Cancer Research, UNITED KINGDOM

## Abstract

The prostate is a male accessory sex gland that produces secretions in seminal fluid to facilitate fertilization. Prostate secretory function is dependent on androgens, although the mechanism by which androgens exert their effects is still unclear. Polyamines are small cationic molecules that play pivotal roles in DNA transcription, translation and gene regulation. The rate-limiting enzyme in polyamine biosynthesis is ornithine decarboxylase, which is encoded by the gene *Odc1*. Ornithine decarboxylase mRNA decreases in the prostate upon castration and increases upon administration of androgens. Furthermore, testosterone administered to castrated male mice restores prostate secretory activity, whereas administering testosterone and the ornithine decarboxylase inhibitor D,L-α-difluromethylornithine (DFMO) to castrated males does not restore prostate secretory activity, suggesting that polyamines are required for androgens to exert their effects. To date, no one has examined polyamines in prostate development, which is also androgen dependent. In this study, we showed that ornithine decarboxylase protein was expressed in the epithelium of the ventral, dorsolateral and anterior lobes of the adult mouse prostate. Ornithine decarboxylase protein was also expressed in the urogenital sinus (UGS) epithelium of the male and female embryo prior to prostate development, and expression continued in prostatic epithelial buds as they emerged from the UGS. Inhibiting ornithine decarboxylase using DFMO in UGS organ culture blocked the induction of prostatic buds by androgens, and significantly decreased expression of key prostate transcription factor, *Nkx3*.*1*, by androgens. DFMO also significantly decreased the expression of developmental regulatory gene *Notch1*. Other genes implicated in prostatic development including *Sox9*, *Wif1* and *Srd5a2* were unaffected by DFMO. Together these results indicate that *Odc1* and polyamines are required for androgens to exert their effect in mediating prostatic bud induction, and are required for the expression of a subset of prostatic developmental regulatory genes including *Notch1* and *Nkx3*.*1*.

## Introduction

The prostate is a secretory exocrine gland that is derived from the embryonic primordium, the urogenital sinus (UGS). The UGS consists of a tube of epithelium surrounded by mesenchyme. Androgens including testosterone and its metabolite dihydrotestosterone act upon androgen receptors in the mesenchyme, which set up a reciprocal signaling cascade between the mesenchyme and epithelium. These signals induce the epithelium to undergo localized thickenings along the epithelium termed prostatic buds. These prostatic buds invade the surrounding mesenchyme where they elongate, branch and canalize to form the ductal network of the mature prostate. Although it is well established that androgens are required to coordinate many molecular events for the precise spatial patterning of the ductal network, the exact mechanisms by which they occur are less clear.

A number of growth factor pathways have previously been shown to either induce or inhibit bud formation. Sonic hedgehog (*Shh*) is not essential for prostatic bud initiation, because *Shh* ^*-/-*^ urogenital sinus tissue cultured in the presence of androgen develop prostatic buds [1–5]. However, inhibiting sonic hedgehog during branching morphogenesis inhibits epithelial cell proliferation. Exogenous Shh also causes mesenchymal expansion [1]. *Bmp4* and *Bmp7* both have an inhibitory role in prostatic bud outgrowth. *Bmp7* null prostates have a more extensively branched prostate compared to control littermates and exert this effect by inhibiting *Notch1* and its target gene, *Hes1* [6]. *Bmp4* also inhibits prostatic bud formation [7]. Crosstalk between the pathways also occurs including regulation of budding and development genes by canonical WNT signaling. Beta-catenin induces *Bmp4* expression and impairs prostatic bud formation [8]. Canonical *Wnt* signaling is also required for proper *Nkx3*.*1* expression [9]. Another member of the Wnt family, Wnt Inhibitory Factor 1 (*Wif1*), is an androgen regulated factor that is expressed in the mesenchyme of the developing urogenital sinus and is more abundant in the male UGS compared to the female UGS [10]. Administering exogenous Wif1 to UGS organ culture increases the number of prostatic buds compared to testosterone alone, demonstrating that Wif1 is functionally required in prostatic bud formation [10]. Clearly prostatic budding results from a complex series of interactions between multiple signaling pathways.

*Nkx3*.*1* is a transcription factor and marker of prostatic bud formation. It is expressed in the areas of the urogenital epithelium that are destined to undergo thickening and elongation to become prostatic buds [11]. *Nkx3*.*1* expression also shows androgenic regulation with expression in the male but not female UGS. Furthermore, when UGS tissue is cultured in the presence of testosterone, *Nkx3*.*1* expression increases [12–14]. In the adult castrated mouse, the prostate undergoes involution and *Nkx3*.*1* expression decreases [15]. In the *Nkx3*.*1* ^-/-^ mouse, prostatic budding still occurs, however prostatic branching in the prostate is significantly reduced and secretory protein production is also affected, indicating that although *Nkx3*.*1* is not required for prostatic budding to occur, it is required for subsequent branching and protein secretory activity [12]

Polyamines are small cationic molecules that are important in development. The first and rate-limiting step in polyamine synthesis is catalyzed by the enzyme ornithine decarboxylase which is encoded by the gene *Odc1* [16]. Ornithine decarboxylase catalyzes the conversion of L-ornithine to putrescine [17]. By the co-ordinated action of S-adenosylmethione decarboxylase (SAMDC), spermidine synthase and spermine synthase, putrescine is converted to spermidine then spermine respectively [18–20]. Ornithine decarboxylase activity can be inhibited by D,L-α-difluromethyornithine (DFMO), an irreversible suicide inhibitor [2,3]. Inhibiting ornithine decarboxylase using pharmacologic agents such as DFMO or ablating *Odc1* using a genetic approach renders pregnant mice unable to carry pups to term [21,22]. Treating pregnant mice with DFMO between days 7–9 of gestation and collecting the pups at day 18 of pregnancy results in unviable fetuses [21]. DFMO treatment also decreases plasma progesterone and androstenedione levels in pregnant mice [21]. Ablating ornithine decarboxylase is embryonic lethal, with conceptuses implanting into the uterine wall then dying at E3.5 due to increased apoptosis [22]. Ornithine decarboxylase is required for expansion of the inner cell mass, with *Odc1* ^*-/-*^ blastocysts that were cultured for three days degenerating, whereas *Odc1* ^*+/-*^ blastocysts develop an expanded inner cell mass. [22]. Clearly, studying ornithine decarboxylase in developmental stages after day 3 of gestation is not possible due to embryonic lethality. This problem can be circumvented by employing explant culture techniques and using small molecule inhibitors to suppress critical parts of the polyamine biosynthetic pathway. Culturing embryonic kidney in DFMO decreases the size of the kidney explants and decreases ductal branching [23]. Inhibiting polyamine production in kidney explants also reduces cell proliferation, and changes the expression of a subset of genes involved in kidney morphogenesis. These changes include increased expression of the epithelial markers *c-ret* and *E-Cadherin* and decreased expression of the mesenchymal transcription factor *Pax8* [23]. Polyamines also play a role in odonotogenesis or tooth formation. Inhibiting polyamine biosynthesis in cultured tooth explants slows down differentiation as well as cell proliferation [24]. Together these studies highlight the importance of ornithine decarboxylase and polyamines in mammalian development.

Ornithine decarboxylase protein is expressed in prostatic epithelial cells in the human, mouse and rat [25,26]. The spatial distribution of ornithine decarboxylase protein and the ornithine decarboxylase antizyme was described in the developing mouse embryo, with expression described in the tubules of the developing kidney [27] but the developing prostate was not mentioned in the embryo-wide examination of ornithine decarboxylase or antizyme protein.

Castration of adult rats decreases prostatic concentrations of putrescine, spermine and spermidine, suggesting that ornithine decarboxylase is androgen regulated [28]. Treating rats with DFMO decreases ventral prostate wet weight that is detectable as early as three days [3] and treating for longer significantly decreases ventral prostate wet weight, putrescine, spermine and spermidine levels [3,29,30].

Polyamines are also required for androgen action in prostate maintenance. When mice are castrated, the prostate wet weight of castrated mice is significantly lower compared to prostate wet weight of intact animals, and treatment with androgen significantly increases prostate wet weight. However, when mice are castrated and treated with testosterone and DFMO (T+DFMO), the prostate wet weight is significantly decreased compared to castrated mice treated with testosterone alone [31]. This decrease in prostatic wet weight is also reflected in the histology, with the lumina of the glands in the T+DFMO treated prostate appearing smaller in size compared to the castrated mice treated with testosterone, and similar in size to the glandular lumina in castrated mice [31]. These results suggest that polyamines are required for androgens to induce secretory activity [31].

Previous studies indicate that polyamines are important for androgenic regulation of prostate function and in development. However, to date, no one has examined whether polyamines play a role in prostatic budding, a developmental process that is regulated by androgen. This study examined ornithine decarboxylase expression in the developing prostate, and found that ornithine decarboxylase activity is required for prostatic bud formation to occur. Furthermore, inhibiting ornithine decarboxylase also downregulated a subset of developmental regulatory genes including *Nkx3*.*1* and *Notch1*.

## Methods

### Reagents

Difluoromethylornithine (DFMO) was a kind gift, courtesy of Dr Patrick Woster. DFMO was reconstituted in tissue culture grade water or 1×PBS to a stock concentration of 1M. Antibodies were obtained against E-Cadherin (Invitrogen 13–1900), Ki67 (Abcam 16667), ornithine decarboxylase (Sigma-Aldrich #O1136), p63 (Santa Cruz SC-8341), ΔN p63 (N-16) (Santa Cruz SC-8609), smooth muscle actin (Sigma A5228) and wide spectrum cytokeratin (Dako Z0622). Mouse on Mouse (MOM) kit was obtained from Vector labs (BMK-2202). Secondary antibodies used were biotinylated secondary antibodies against mouse (BA-9200) and rabbit (BA-1000) (Vector), HRP conjugated anti-mouse (SC-2055) (Santa Cruz), and secondary antibodies conjugated to Texas Red fluorophores against rabbit (TI-1000) and goat (TI-5000) (Vector). For visualizing E-Cadherin in wholemount UGS cultures and ornithine decarboxylase using immunofluorescence, streptavidin conjugated to Alexafluor-488 (S32354 Invitrogen) was used.

### Tissue collection

E16 (embryonic day 16) timed CD-1 or C57/Bl6 pregnant mice were obtained from Charles River. All animals were euthanized using carbon dioxide then cervical dislocation. Fetuses were removed from the uterus and sexed according to the presence of testes or ovaries. UGS tissues were isolated from fetuses using a pair of 30 gauge needles as described previously [32]. Tissues were either cultured, fixed in 4% paraformaldehyde overnight at 4°C for immunofluorescence and immunohistochemistry, or snap frozen in liquid nitrogen for gene expression studies. Adult male mice (8 weeks or older) were also obtained from Charles River. Prostates were excised, fixed in 4% PFA overnight at 4°C then processed for immunohistochemistry.

All animal experimentation in this study was conducted in accord with accepted standards of humane animal care as outlined in the NIH Guide for the Care and Use of Laboratory Animals. Experimentation was approved by the University of Wisconsin-Madison Institutional Animal Care and Use Committee (#M022070).

Human prostate samples were obtained from radical prostatectomy procedures carried out at the University of Wisconsin-Madison. The University of Wisconsin Institutional Review Board (IRB) (2012–1033, 2012–0508) approved retrospective review of patient information and demographics included in this study and waived the need for written informed consent from patients. Tissues were obtained from a pathology archive and used for diagnostic purposes; therefore patient consent was not required.

### UGS epithelial and mesenchymal separation

To examine *Odc1* mRNA abundance in the mesenchyme and epithelium in the UGS, we separated the mesenchyme and epithelium as has been described previously [32]. Briefly, UGS tissues from E16 and E18 male embryos were isolated as described above. The tissues were digested in 1% trypsin at 4°C for 75 minutes, then the reaction was terminated with 10% fetal bovine serum in DMEM. To eliminate stickiness due to the release of genomic DNA, several grains of DNase were added. The tissue compartments were separated using two pairs of fine forceps and a dissecting scope. The tissue compartments of several UGS tissues were pooled then snap frozen for subsequent RNA isolation, cDNA synthesis and QPCR analysis.

### UGS culture

To study the effect of inhibiting ornithine decarboxylase *in vitro*, E16 UGS tissues were cultured for six days in the presence of difluoromethylornithine (DFMO). Basal culture media consisted of phenol-red free DMEM/Ham’s-F12, supplemented with 1×ITS (insulin-transferrin-selenium) and 0.1% antibiotic/anti-mycotic. Tissues were cultured on pieces of membrane (Millipore) and floated on media. To induce prostatic budding, tissues were treated with 10nM of testosterone. To examine the effect of ornithine decarboxylase inhibition, tissues were treated with a combination of testosterone (final concentration 10nM) and DFMO (final concentration 10mM). Media was changed every 48 hours. To ensure consistency, tissues from one sex were used for direct comparisons during analysis, however there was no difference in treatment between male and female tissues. After six days, tissues were fixed in 4% PFA for immunofluorescence and immunohistochemistry. For immunofluorescence, fixed tissues were dehydrated in an increasing methanol series to 100% methanol and stored at -20°C until required. For immunohistochemistry, fixed tissues were stored in 70% ethanol until required, and processed for histology into paraffin. For gene expression studies, tissues were snap frozen in liquid nitrogen. Pools of three to five tissues were snap frozen together to obtain enough RNA for gene expression studies. At least three pools of tissues were obtained for each treatment group.

### Q-PCR

RNA was extracted from UGS pools using total RNA extraction kit (Takara-Clonetech) according to manufacturer’s instructions. RNA (1000ng) was synthesized into cDNA using RNA to cDNA Eco-dry Premix cDNA synthesis kit (Takara-Clonetech) according to manufacturer’s instructions. The resulting cDNA synthesis reaction was diluted to a final concentration of 10ng/μl. Primers were designed spanning exon-exon boundaries of genes (see S1 Table). Each primer set was optimized to obtain an amplification efficiency between 90–110%. QPCR was carried out using 2 × Power Sybr green reaction mix (Life Technologies), and 50–200nM final concentration of primer and 10ng of diluted cDNA. Reactions were carried out on Applied Biosciences thermocycler. The QPCR conditions were as follows: 95°C for 10 minutes, then 40 cycles at 95°C for 15 seconds and 60°C for 1 minute, then subsequent dissociation curve analysis. All expression values were normalized to the endogenous control gene *Hprt1* or *Actb*. Raw Ct values were converted to relative expression values by calculating 2^-(Ct of gene of interest-Ct of control gene or ΔCt)^. Cultured UGS relative expression was calculated using the 2^([Ct of gene of interest-Ct of control gene]- [ΔC of treatment – ΔC of control] or ΔΔCt)^ comparing everything to control treatment.

### Histology and Immunohistochemistry

Tissues were fixed in 4% PFA for a minimum of 16 hours at 4°C. They were washed in an increasing graded methanol series and stored in 100% methanol where they were stored at -20° Celsius until required. Before processing for histology, tissues were rehydrated from a graded methanol series back to 1 × PBS. To assist with tissue orientation, UGS tissues were embedded in 1% agar. Tissues were processed for histology and immunohistochemistry and embedded in paraffin. Sections (6μm) were cut on a microtome, floated on a 37°C waterbath and mounted on Superfrost slides. They were dried overnight in a 37°C oven, then stored at room temperature until use. To examine histological morphology, tissues were stained with hematoxylin and eosin, according to standard protocols.

To examine protein distribution in the cultured UGS, antibodies against cytokeratin, smooth muscle actin and p63 were used. Conditions for each antibody are listed S2 Table. For antibodies raised in mouse (p63 and SMA), the Mouse on Mouse (MOM) kit was used to visualize staining (Vector labs). Sectioned tissues were dewaxed in Citrosolv (Fisher Scientific) and rehydrated through a decreasing ethanol series through to water. Sections were then permeabilized in 0.1% Triton X-100 in 1 × TBST [0.1M Tris, 0.15M NaCl, pH 7.5 with 0.05% Tween-20] for 15 minutes at room temperature with shaking. Endogenous peroxidase activity was quenched with 3% hydrogen peroxide in 100% methanol for 15 minutes at room temperature with shaking. Tissues were briefly rinsed with 1×TBS, then antigen retrieval was carried out in Vector Antigen unmasking solution diluted 1:100 in water, preheated to 95°C. Slides were immersed in preheated antigen retrieval solution for 30 minutes then cooled at room temperature for a minimum of 20 minutes. The tissues were washed for 3×5 minutes in 1×TBS. They were blocked in blocking buffer for one hour at room temperature (see S2 Table). The primary antibody was applied and the slides incubated at 4°C for 18 hours. Primary antibody was tapped off and the slides were washed 3×5 minutes in 1×TBST. Biotinylated secondary antibody (Vector) diluted 1:250 was applied to the tissues and incubated for one hour at room temperature. The sections were washed 3×5 minutes in TBST and ABC complex (Vector) was applied and incubated for 30 minutes at room temperature. The tissues were washed in 3×5 minutes TBST then color was developed using diaminobenzidine activated with hydrogen peroxide (Vector). When the desired staining was obtained, the tissues were washed in water to stop the reaction. They were counterstained with Harris hematoxylin, dehydrated through an increasing graded ethanol series and Citrosolv and mounted in Permount mounting reagent (Fisher-Scientific). Ki67 immunohistochemistry was carried out as described previously [33].

To examine ornithine decarboxylase distribution in the human and adult mouse prostate, we used a visualization system from Biocare Medical. Slides were dewaxed in Citrosolv and rehydrated as described above. Antigen retrieval was carried out in a Diva Decloaker antigen retrieval system using preset factory settings. Slides were cooled and rinsed in deionized water. They were washed in 1×TBST for two minutes with shaking then samples were blocked in Rodent Block M (Biocare Medical, RBM9616) for 30 minutes at room temperature in a humidified chamber. The slides were washed twice in 1×TBS for 2 minutes, then incubated in ODC1 primary antibody (Sigma, O1136), diluted 1:100 in Da Vinci green universal diluent (Biocare Medical, PD900H) for 1 hour at room temperature in a humidity chamber. The slides were washed in 1×TBST twice for two minutes then the sections were incubated in Mach-2 mouse secondary antibody conjugated to alkaline phosphatase (Biocare Medical, MALP521G) for 30 minutes at room temperature in a humidified chamber. The slides were washed twice in 1×TBST for two minutes, then visualization was carried out using Warp Red chromagen kit (Biocare Medical, WR806H) according to manufacturer’s instructions, incubating the chromagen for five minutes. The slides were finally rinsed in deionized water, counterstained in haematoxylin, dried at 60°C for 15 minutes, taken through Citrosolv and mounted in permount. A minimum of three animals per stage and sex was stained for each antibody.

### Odc1 and Vimentin/p63 immunofluorescence

To examine ornithine decarboxylase co-localization with vimentin in the developing urogenital sinus, we visualized these proteins using immunofluorescence. Tissues were dewaxed and rehydrated as described above. They were permeabilized in 0.1% Triton X-100 in TBST, then immersed in 3% hydrogen peroxide in absolute methanol to quench endogenous hydrogen peroxide activity as described above. The tissues were rinsed in 1×TBS then immersed in Vector Antigen Retrieval Solution (Vector; diluted 1:100 in water and preheated to 95°C) for 30 minutes. The slides were cooled for 20 minutes then washed three times for five minutes each wash in 1×TBST. The tissues were blocked using MOM block for 1 hour at room temperature. Primary antibody diluted in MOM diluent (Ornithine decarboxylase diluted 1:100, p63 diluted 1:100, Vimentin diluted 1:50) was applied to sections and incubated overnight at 4°C. During the second day, all washing and incubation steps were conducted in darkened and humidified chambers. The following morning, the slides were washed three times in 1×TBST for 5 minutes each wash. Secondary antibodies (goat anti-mouse conjugated to HRP and goat anti-rabbit or rabbit anti-goat conjugated to Texas Red) were diluted 1:250 in antibody dilution buffer (1% blocking reagent [Roche], 0.5% normal goat serum, 1% BSA in 1×TBST), and applied for 1 hour at room temperature. The tissues were washed three times in 1×TBST for 5 minutes each wash. Tyramide conjugated to biotin and diluted 1:333 in 0.01% hydrogen peroxide was applied to the slides and incubated at 15 minutes at room temperature. The tissues were washed three times in 1×TBST for five minutes each wash. Streptavidin conjugated to Alexafluor-488 diluted 1:500 in antibody dilution buffer was applied for 1 hour at room temperature. The tissues were washed three times in 1×TBST for five minutes each wash, then the tissues were mounted in Ultra Cruz Mounting media with DAPI (Santa Cruz SC-24941). Sections were imaged using confocal microscopy, with red corresponding to vimentin or p63 expression (as indicated in figure), green corresponding to ornithine decarboxylase expression and blue corresponding to DAPI nuclear stain.

### Wholemount immunofluorescence

Wholemount UGS tissues were rehydrated in a graded methanol series to PBS, washing in each solution for 10 minutes. The tissues were blocked in 2 × 1 hour washes of 5% normal rabbit serum in 1×PBS. They were incubated in E-cadherin antibody (Invitrogen 13–1900) diluted 1:1000 in 5% rabbit serum at 4°C overnight. The tissues were washed in 5 × 1 hours in 5% rabbit serum then incubated in biotinylated rabbit anti-rat secondary antibody diluted 1:250 in 5% rabbit serum overnight. The tissues were again washed in 5 × 1 hours in 5% rabbit serum and incubated in streptavidin conjugated to Alexafluor-488 diluted 1:600 in 5% rabbit serum overnight. The tissues were washed in 2 × 10 minutes in 1XPBS then the tissues were stored in Ultra Cruz mounting medium with DAPI (Santa Cruz SC-24941) until they were imaged. The tissues were mounted, coverslipped and imaged using confocal microscopy. The tissues were imaged using Z-stack acquisition and the images compressed to form a 2D representation of the 3D stack. Two individuals blinded to the treatment conditions counted the number of buds and the data was averaged. A minimum of five animals were counted per treatment group.

### Statistical analysis

All relative expression values of genes in the UGS were averaged. Comparisons between different treatments were compared using one-way Anova with Tukey’s test for multiple comparisons in the software package R version 2.15.1 for Mac [34], using the R commander graphical user interface [35]. Differences were considered statistically significant when p<0.05.

## Results and Discussion

Previous work has shown that, in the adult prostate, ornithine decarboxylase activity is androgen-dependent. *Odc1* mRNA decreases and increases with androgen withdrawal and administration respectively in rats, with intact rat prostates expressing *Odc1* mRNA, castrated rat prostates showing little mRNA accumulation, and increased mRNA accumulation after testosterone administration [30,36]. In adult mouse prostate, ornithine decarboxylase activity parallels *Odc1* mRNA accumulation, with activity decreasing to undetectable levels in castrated males, and increasing significantly upon injection of androgen [37]. In the rat neonate, ornithine decarboxylase in the prostate increases significantly at two weeks and coincides with a significant increase in prostate wet weight, DNA and protein [38]. The *Odc1* promoter contains an androgen-responsive element (ARE), that when mutated *in vitro*, loses its transactivational activity [36]. For these reasons, we were interested whether ornithine decarboxylase activity is similarly androgen regulated during early prostate development, another androgen-dependent process.

### Ornithine decarboxylase protein was expressed in the epithelium of human and mouse adult prostate

**Fig 1:**
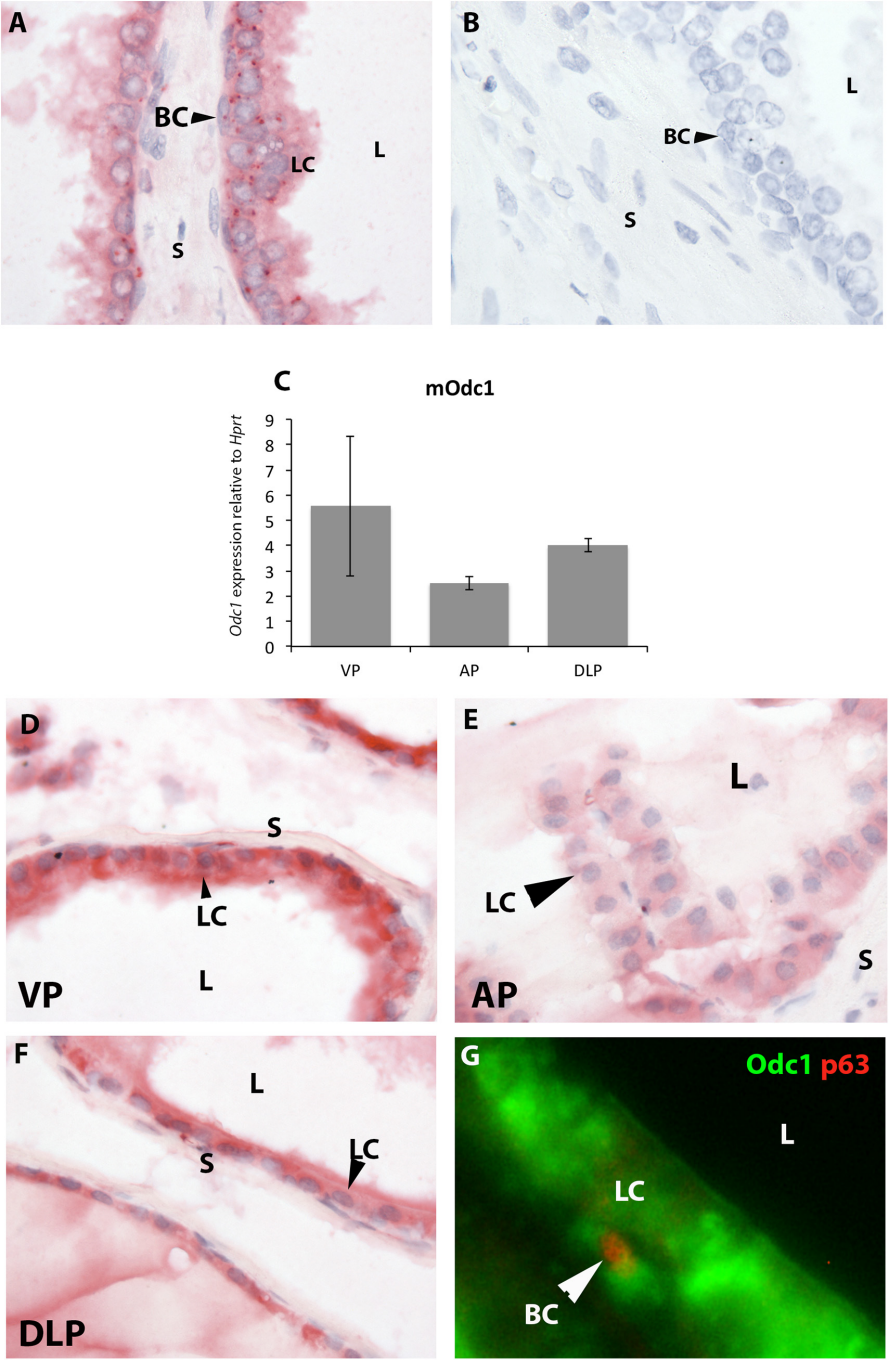
Ornithine decarboxylase expression in the human and mouse adult prostate. In the adult human prostate, ornithine decarboxylase protein was expressed in the basal and luminal cells of the epithelium (A). The negative control with primary antibody omitted showed no staining (B). In the mouse adult prostate, *Odc1* mRNA was expressed in the VP, DLP and AP (C), and there was no statistically significant difference in mRNA levels among the lobes. *Odc1* mRNA levels were normalized to *Hprt*. Ornithine decarboxylase protein was expressed in the epithelium of the VP (D), AP (E) and DLP (F). Using immunofluorescence, we observed ornithine decarboxylase staining (green) in close proximity to p63 staining (red), suggesting that ornithine decarboxylase was expressed by present basal as well as luminal epithelial cells (G). Immunostaining for A, B, D-F is in pink. Images were taken at 100X magnification unless otherwise stated. Abbreviations used: BC basal cells, L lumen, LC luminal cells, S stroma.

In the human prostate, ornithine decarboxylase protein was expressed in the cytoplasm of the epithelium in both basal and luminal epithelial cells (Fig 1A), consistent with previous studies [25,26]. The negative control, with primary antibody omitted, showed no staining (Fig 1B). Previous work has shown that ornithine decarboxylase protein is expressed in the epithelium of the mouse prostate, however the prostate lobe examined was not specified [25]. To further characterize ornithine decarboxylase expression in the mouse prostate, we examined *Odc1* mRNA levels in the different lobes of the mouse prostate (ventral prostate VP, anterior prostate AP and dorsolateral prostate DLP). We found that *Odc1* mRNA was expressed in all three lobes and they did not differ significantly in *Odc1* mRNA levels (Fig 1C). We also observed ornithine decarboxylase protein in the prostate epithelium in the VP, DLP and AP (Fig 1D, 1E and 1F), which is consistent with previous work [25].

The similarity of ornithine decarboxylase localization in the epithelium of both the mouse and human prostate suggests that spatial expression and likely function of ornithine decarboxylase are conserved between the human and the mouse prostates.

### Ornithine decarboxylase mRNA levels and protein distribution in the developing urogenital sinus

It is well established that ornithine decarboxylase expression and activity is under androgenic control in the adult prostate. We then asked whether androgens in the male during prostate organogenesis induced the expression of ornithine decarboxylase, which in turn induced the initiation of prostatic bud formation. Since prostatic budding only occurs in males and not in females, we examined *Odc1* mRNA abundance in the male and female UGS from E15 to E18, encompassing the period of prostatic budding.

**Fig 2:**
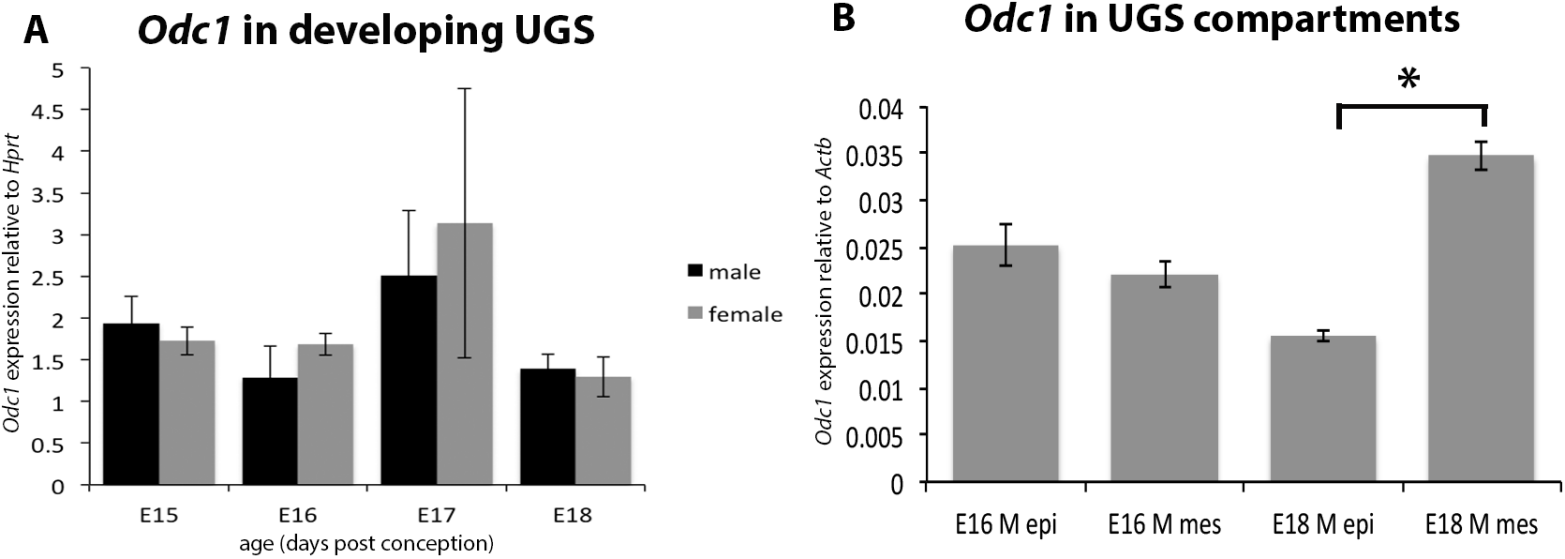
*Odc1* mRNA levels in the developing urogenital sinus and in separated UGS tissue compartments. *Odc1* transcripts were present in the male and female UGS throughout the period of prostatic bud induction from E15-E18, although they did not vary between males and females during this period (A). *Odc1* transcript abundance did not differ between the epithelium and mesenchyme at E16 (B), although there were significantly more *Odc1* transcripts in the mesenchyme compared to the epithelium at E18 (B). Abbreviations used: epi epithelium, mes mesenchyme. *, p<0.05.

We assessed the mRNA levels of *Odc1* in the developing UGS in both male and female mice during embryogenesis using quantitative PCR (qPCR). *Odc1* mRNA was expressed in both the male and female UGS during the period when prostatic buds are forming from embryonic day 15 to embryonic day 18 (Fig 2A). However *Odc1* mRNA levels did not differ between males and females during the period of prostatic bud formation (Fig 2A).

We also examined *Odc1* mRNA levels in the epithelium and mesenchyme of the developing male urogenital sinus at E16 before budding has commenced and at E18, after prostatic budding has commenced (Fig 2B). At E16, there was no significant difference in *Odc1* mRNA abundance between the epithelium and mesenchyme (Fig 2B). However, at E18 after prostatic budding has commenced, there was significantly more *Odc1* mRNA in the mesenchyme compared to the epithelium (Fig 2B).

**Fig 3:**
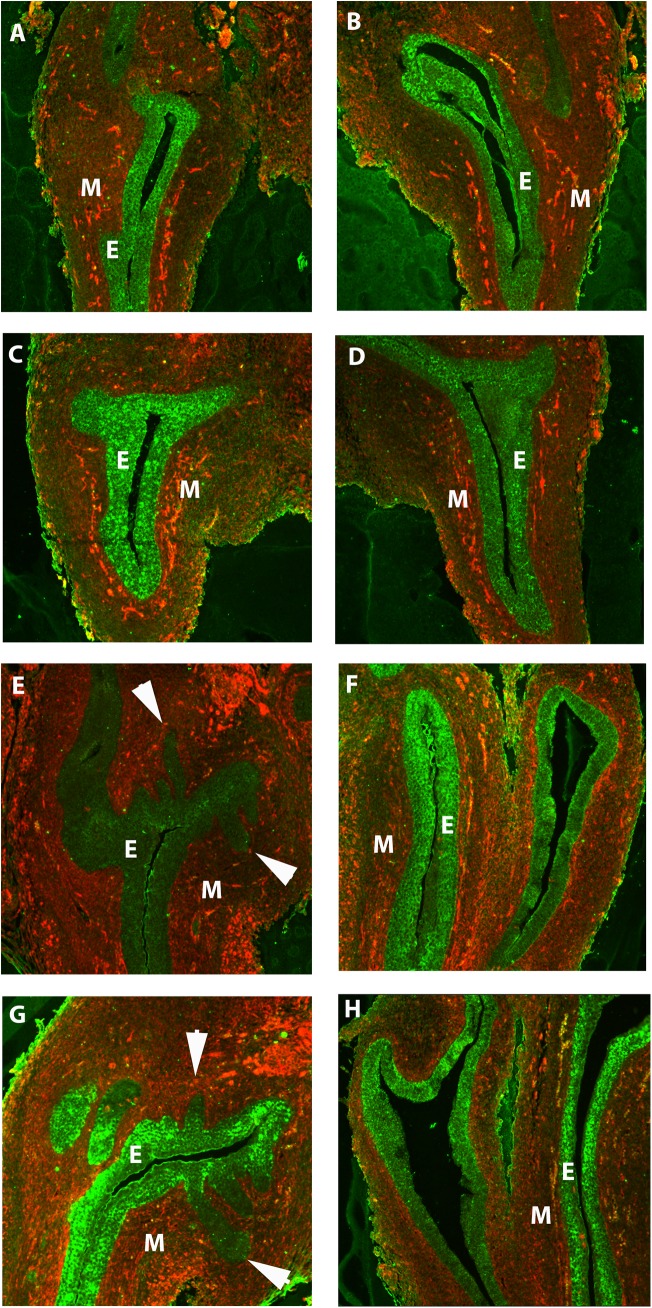
Ornithine decarboxylase protein was present in the urogenital epithelium in the developing urogenital sinus. In the male UGS before budding at E15 (A) and E16 (B), ornithine decarboxylase protein (green) was present in the epithelium. As the buds emerged from the epithelium at E17 (E) and E18 (G), ornithine decarboxylase protein was still expressed in the epithelium and buds. In the female UGS, ornithine decarboxylase was present in the epithelium at E15 (B) and E16 (D), the period before prostatic buds are initiated in the male UGS. At E17 (F) and E18 (H), ornithine decarboxylase protein continued to be expressed in the urogenital epithelium. Ornithine decarboxylase staining is in green, and vimentin staining is in red. There was some co-localization of ornithine decarboxylase and vimentin (yellow) in the mesenchyme. Arrowheads denote prostatic buds. Abbreviations: E epithelium, M mesenchyme.

We then assessed the expression of ornithine decarboxylase protein in the developing UGS using immunofluorescence, and compared male and female tissues at similar stages. We used vimentin to distinguish mesenchymal tissue (red) from the urogenital epithelium (Fig 3). Ornithine decarboxylase protein (green) was expressed in the UGS in both the male (Fig 3A, 3C, 3E and 3G) and the female (Fig 3B, 3D, 3F and 3H). In the male UGS, ornithine decarboxylase protein was present in the urogenital epithelium before budding was apparent at E15 (Fig 3A) and E16 (Fig 3C). By E17, prostatic epithelial buds were apparent, and projecting into the mesenchyme (Fig 3E, arrows), and ornithine decarboxylase staining was expressed in the urogenital epithlium as well as the buds. By E18, ornithine decarboxylase protein was still expressed in the urogenital and developing prostatic epithelium (Fig 3G, arrows). In the female UGS, the epithelium remained smooth with no apparent signs of prostatic budding (Fig 3B, 3D, 3F and 3G). Similar to the male UGS, ornithine decarboxylase protein was expressed in the urogenital epithelium (Fig 3B, 3D, 3F and 3G). In all stages, there was some ornithine decarboxylase staining co-localized with vimentin (yellow), indicating that there was some ornithine decarboxylase in the mesenchyme. The negative control (with primary antibody omitted) showed no staining (data not shown).

The lack of sexual dimorphism in *Odc1* mRNA levels and the presence of ornithine decarboxylase protein in the female urogenital sinus suggested that androgen was not required to induce ornithine decarboxylase expression, which is different than the clear androgenic regulation of the ornithine decarboxylase in the adult mouse prostate [3,30]. Furthermore, ornithine decarboxylase protein was expressed in the male UGS before the onset of bud formation, further supporting the argument that at this stage of development, ornithine decarboxylase expression in the developing UGS is independent of androgens and that androgen responsiveness of ornithine decarboxylase is likely acquired after the prostate has matured in adulthood.

### Culturing the UGS in DFMO inhibited testosterone-induced prostatic bud formation

In the embryonic UGS, androgens do not induce ornithine decarboxylase expression, so the next question is whether ornithine decarboxylase activity is required for androgens to induce its effect on prostate bud formation? To address this question, we undertook an organ culture approach using testosterone and DFMO (difluoromethylornithine). DFMO is a non-reversible inhibitor of ornithine decarboxylase [4] and has been used to inhibit ornithine decarboxylase in organ culture of embryonic kidney [23] and teeth [24]. In initial studies, we did a dose response of DFMO in UGS culture with testosterone, and found that 10mM was the lowest dose required to reduce prostatic budding, which is similar to the inhibitory dose used in other *in vitro* organ culture studies [23].

**Fig 4:**
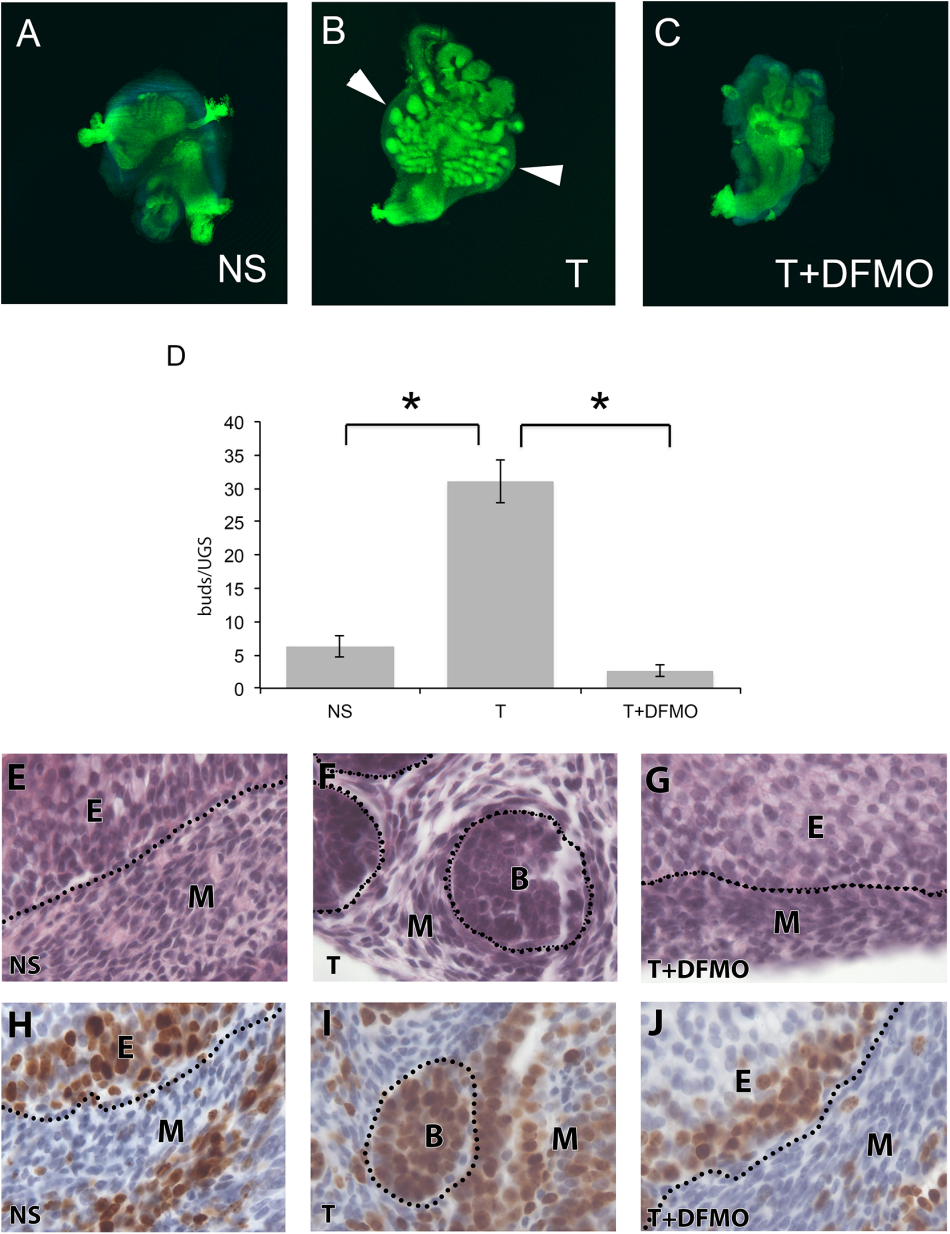
E16 UGS cultured in the presence or absence of testosterone and DFMO. The tissues were fixed in 4% PFA then stained for E-Cadherin to visualize the urogenital epithelium. In the no steroid control media, the urogenital epithelium remained smooth with no evidence of prostatic bud formation (A). After six days of treatment with testosterone, the UGS underwent prolific bud formation (B). Culturing the UGS with testosterone and DFMO decreased the number of prostatic buds compared to the testosterone treatment, and comparable to the no steroid control UGS (C). For each treatment, the numbers of buds were counted. Testosterone induced significantly more buds compared to no steroid treatment, and treating UGS tissues with DFMO and testosterone significantly decreased the number of buds compared to testosterone alone (D). Culturing the UGS for six days did not adversely affect the histology of cells regardless of whether they were cultured in NS media (E), T (F) or T+DFMO (G) media. In all three treatments, the nuclei were large and healthy looking with no evidence of pyknosis. To assess proliferation, we assessed Ki67 expression in our UGS culture. Ki67 was expressed in specific epithelial cells and mesenchymal cells in the NS control UGS (H). In the T treated UGS, Ki67 was expressed in cell clusters in prostatic buds, as well as specific cells within the mesenchyme (I). In T+DFMO treated UGS, Ki67 was expressed in certain cells in the epithelium as well as the mesenchyme. Abbreviations: NS no steroid control, T testosterone, DFMO difluoromethylornithine. The dotted line demarcates the border between epithelium and mesenchyme. Images E-J were taken at 100X magnification.

In the no steroid (NS) control, the UGS tissues did not undergo epithelial bud growth (Fig 4A). The tube of epithelium was smooth and of uniform thickness. When embryonic urogenital sinus tissue was cultured in testosterone, prostatic buds grew from the epithelium and into the overlying mesenchyme (Fig 4B) [39]. When prostatic buds were counted, testosterone treatment significantly increased the number of prostatic buds produced compared to the no steroid control, (Fig 4D, * p<0.05). However, when the UGS was cultured in testosterone with DFMO, prostatic budding was extremely reduced compared to the testosterone treatment (Fig 4C). When the numbers of buds were counted, testosterone and DFMO treatment significantly decreased the number of prostatic buds compared to testosterone treatment only (Fig 4D, *<0.05) and the number of buds in the T+DFMO treated UGS were comparable to those in the NS control.

After 6 days in culture, the UGS tissues appeared healthy. Tissues in all the different treatments underwent periodic peristaltic contractions, indicating that the various treatments did not induce toxicity during the culture period [40]. Furthermore, the histology (H&E stain) showed that the 6-day culture period and the DFMO did not induce toxicity effects (Fig 4E, 4F and 4G). In all treatments, the cells looked healthy with clear large nuclei and had no evidence of overt pyknosis (Fig 4E, 4F and 4G).

Polyamines are required for DNA replication, so we initially hypothesized that polyamines are required for overall growth. Ki67 is a nuclear protein that is expressed in all phases of the cell cycle except G0 phase when cells are quiescent, and thus is a marker for cellular proliferation [41,42]. To examine cellular proliferation, we visualized Ki67 expression in the cultured UGS. In the NS treated UGS, Ki67 expression was primarily in the basally located epithelial cells (Fig 4H). In the testosterone treated UGS, Ki67 was concentrated in the distal tips of the prostatic buds, with some cells also expressing Ki67 in the mesenchyme (Fig 4I). Ki67 expression in the testosterone treated UGS suggested that the distal tips of the prostatic buds were highly proliferative. In the T+DFMO treated UGS, Ki67 was expressed mainly in the basally located epithelial cells (Fig 4J), with some scattered cells also staining in the mesenchyme suggesting that DFMO did not inhibit global cell proliferation.

Previous studies indicate that reduced branching in metanephros organ culture due to DFMO can be rescued with the addition of putrescine [23], or spermine and spermidine in tooth organ culture [24]. In our own studies, we attempted to rescue prostatic budding by adding similar concentrations of putrescine, spermine and spermidine to those published previously [23,24], but we did not observe a rescue of prostatic bud formation. We then added varying amounts of putrescine, spermine and spermidine to organ culture and still did not observe rescue of prostatic budding. This could be due to difficulty in polyamines entering cells throughout the cultured organs and/or a requirement that polyamines be localized during prostatic budding rather than generally present throughout the UGS.

Together these results suggest that polyamines are required for androgens to induce bud formation. Polyamines are required in other organs that undergo budding, branching and elongation such as neurons, lung and kidney [23,43,44]. Neurons that are derived from embryonic brain and cultured at low density decrease in number over time, whereas the addition of spermidine significantly increases the number of surviving neurons as well as causes significant elongation of axons compared to control [44]. When embryonic chicken lungs are cultured in the presence of DFMO for three days, they develop significantly fewer bronchi compared to control cultures [43]. Finally, when embryonic kidney is cultured in the presence of DFMO, fewer branches form compared to the control [23]. Together all these data suggest that polyamines may form a part of the general bud outgrowth program.

### Testosterone and DFMO treatment did not change the transcription of enzymes that regulate polyamine levels

**Fig 5:**
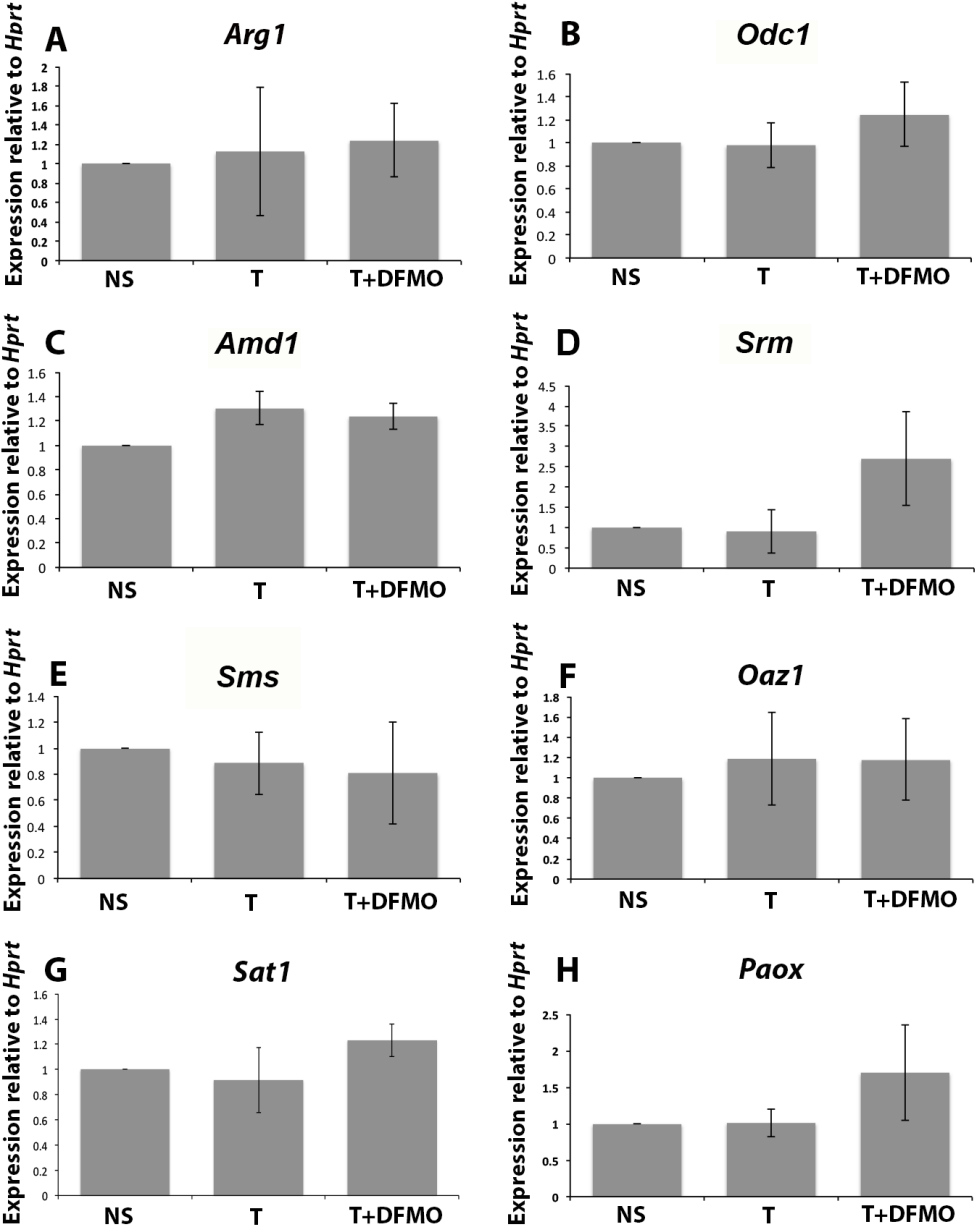
mRNA levels of polyamine biosynthetic and catabolic enzymes in the cultured UGS. The mRNA levels of biosynthetic enzymes *Arg1* (A), *Odc1* (B), *Amd1* (C), *Srm* (D) and *Sms* (E) did not change in response to treatment with testosterone or T+DFMO. Similarly, the regulatory enzymes *Oaz1* (F), *Sat1* (G) and *Paox* (H) were also unchanged in response to testosterone or T+DFMO.

In the previous section, we showed that inhibiting polyamine biosynthesis reduces prostatic budding. We then asked whether the presence of androgens and/or inhibiting polyamine biosynthesis in the UGS affected the transcription of polyamine biosynthetic or catabolic enzymes. We cultured the UGS for six days in no steroid media (NS), testosterone supplemented media (T) or testosterone and DFMO supplemented media (T+DFMO), then snap froze the tissue for downstream gene analysis. Culturing the UGS in testosterone did not induce mRNA transcription of biosynthetic enzymes arginase (*Arg1*, Fig 5A), ornithine decarboxylase (*Odc1*, Fig 5B), S-adenosylmethionine decarboxylase (*Amd1*, Fig 5C), spermidine synthase (*Srm*, Fig 5D) and spermine synthase (*Sms*, Fig 5E). Similiarly, culturing the UGS in T+DFMO did not alter mRNA expression levels of any of the polyamine biosynthetic enzymes examined (Fig 5A–5E).

Polyamine levels are tightly regulated by a balance of biosynthesis and catabolism, so we also examined mRNA of regulatory enzymes. Culturing the UGS in testosterone did not induce transcription of ornithine antizyme (*Oaz1*, Fig 5F), Spermine/Spermidine N-acetyltransferase (*Sat1*, Fig 5G) or polyamine oxidase (*Paox*, Fig 5H). Similar to the biosynthetic enzymes, there was no change in the transcription of these enzymes in response to T + DFMO (Fig 5F–5H).

Several polyamine biosynthetic enzymes have been shown to be under androgenic regulation such as ornithine decarboxylase in the prostate, skeletal muscle and kidney, and S-adenosylmethionine decarboxylase (SAMDC) in the kidney. Abundance of mRNA of polyamine biosynthetic enzymes such as *Odc1*, *Amd1*, *Srm* and *Sms* did not change in the presence of testosterone or in the presence of T + DFMO (Fig 5). When testosterone is administered to mice, *Odc1* mRNA and protein expression increases in the mouse kidney [45]. In the mouse prostate and seminal vesicles, *Odc1* mRNA accumulates in the epithelial cells upon androgen administration [45]. SAMDC, which is encoded by *Amd1*, is similarly androgen regulated in the adult mouse prostate, seminal vesicle and kidney [45]. Both *Amd1* and *Odc1* promoters contain androgen response elements but in the developing UGS, they did not appear to have acquired sensitivity to androgen at the mRNA level (Fig 5B and 5C).

### Inhibiting polyamine production did not interfere with compartmentalization of the cells in the developing urogenital sinus

Inhibiting polyamine activity clearly inhibited prostatic bud growth even in the presence of androgen, so we asked whether this was due to a disturbance in cellular identity in the UGS. To answer this question, we examined the distribution of proteins that demarcate areas of the urogenital sinus such as cytokeratins and p63 in epithelial cells and smooth muscle actin (SMA) in the mesenchyme.

**Fig 6:**
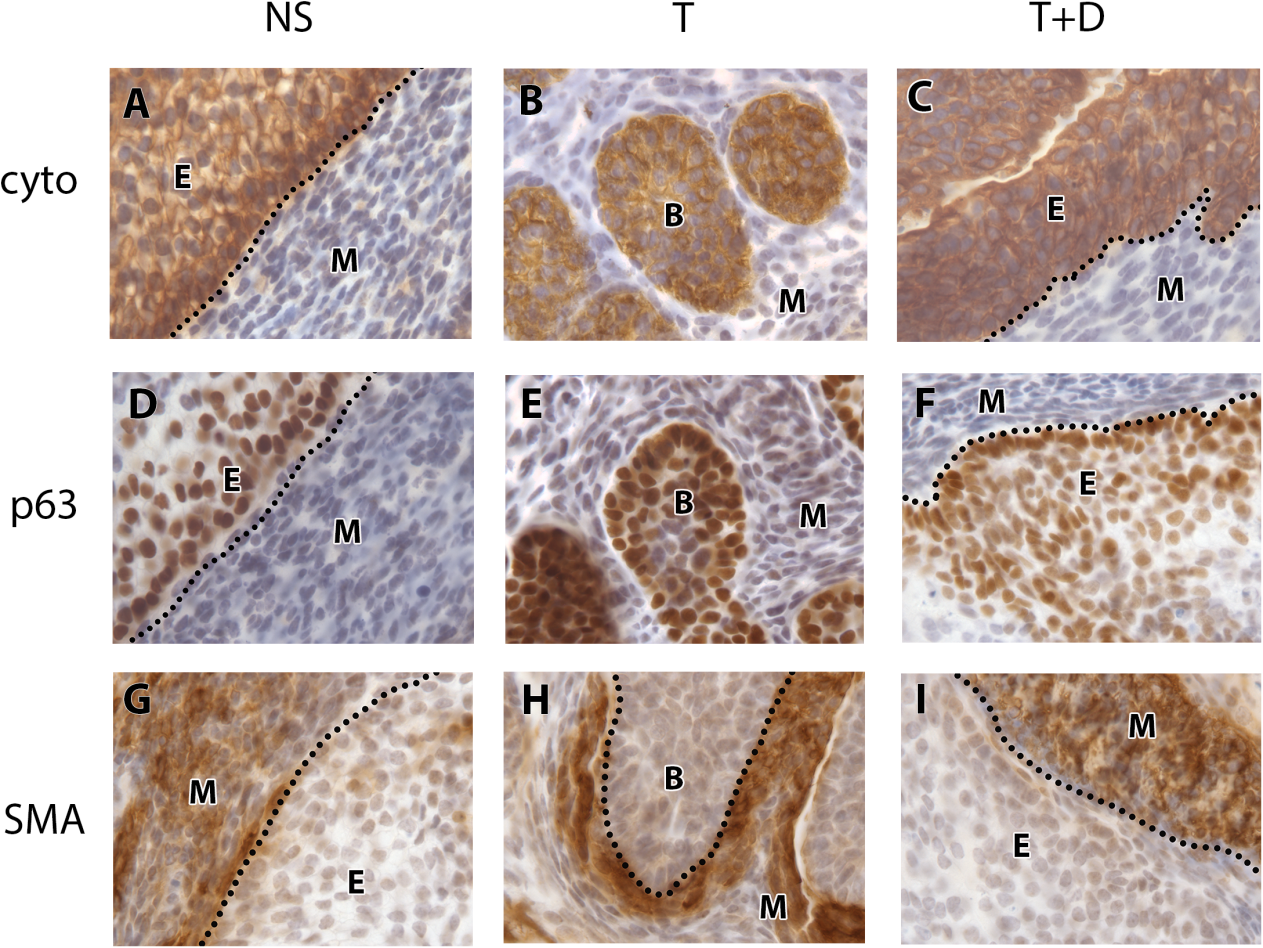
Markers of tissue compartments in the cultured UGS. Cytokeratin staining was confined to cytoplasm of the urogenital epithelial cells in the NS control (A), T treated UGS (B) and in the T+DFMO treated UGS (C). p63 staining was present in the nuclei of epithelial cells in the NS control (D), T treated UGS (E) and in the T+DFMO treated UGS (F). SMA staining was present in the cytoplasm of peri-ductal mesenchymal cells in the NS control (G), T treated UGS (H) and T+DFMO treated UGS (I). Positive immunostaining is brown. The dotted line demarcates the boundary between epithelium and mesenchyme. Abbreviations: B prostatic bud, cyto cytokeratin, DFMO difluoromethylornithine, E epithelium, M mesenchyme, NS no steroid control, SMA smooth muscle actin, T testosterone.

Using immunohistochemistry, we showed that the urogenital epithelium expressed cytokeratins. In the NS treated UGS, cytokeratins were expressed in the urogenital epithelium, which remained smooth after six days in culture media (Fig 6A). In the testosterone treated UGS, cytokeratins were expressed in all the cells of the urogenital epithelium, which underwent extensive bud formation (Fig 6B). In the T+DFMO treated UGS, cytokeratins were still expressed in the epithelium, which did not undergo prostatic budding (Fig 6C). Under all conditions, cytokeratins were always expressed in the epithelium, suggesting that polyamines did not alter cytokeratin expression in epithelial cells.

We also examined p63 expression, another marker for the epithelium. In the NS treated UGS, p63 protein was expressed in the nuclei of epithelial cells (Fig 6D). In the T treated UGS, p63 protein was expressed in nuclei of epithelial cells lining the prostatic buds (Fig 6E). In the T+DFMO treated UGS, p63 protein was also expressed in the nuclei of epithelial cells which did not undergo prostatic bud formation (Fig 6F). Under all culture conditions, p63 expression was only expressed in the epithelium.

To examine the mesenchyme in the developing UGS, smooth muscle actin (SMA) was examined. In the NS treated UGS, SMA was expressed in the cytoplasm of mesenchymal cells (Fig 6G). In the T treated UGS, SMA was expressed in the cytoplasm of periductal mesenchymal cells, surrounding the prostatic bud tips (Fig 6H). In the T+DFMO treated UGS, SMA was expressed in the cytoplasm of mesenchymal cells (Fig 6I). Under all culture conditions, SMA expression remained in the mesenchyme of the UGS, irrespective of culture treatment.

In summary, although polyamine depletion affected prostatic bud formation, it did not affect the cellular identity of different compartments within the UGS, with all epithelial cells expressing cytokeratins and p63 and mesenchymal cells expressing SMA. One of the caveats associated with using an organ culture model is that although growing UGS tissues *in vitro* does induce prostatic budding, it fails to recapitulate epithelial and stromal differentiation [46]. Previous work has shown that when UGS tissues are grown in culture for up to 21 days, although prostatic ducts form, cytodifferentiation fails to occur [46]. p63 expression in the developing male urogenital sinus is localized throughout the epithelium from E14-E18, which is a reasonable comparison to our current organ culture study [47]. Thus in this current study, we show that polyamine depletion through the inhibition of ornithine decarboxylase inhibits prostatic bud formation.

### Inhibiting ornithine decarboxylase reduces the induction of a subset of prostatic developmental regulatory genes

**Fig 7:**
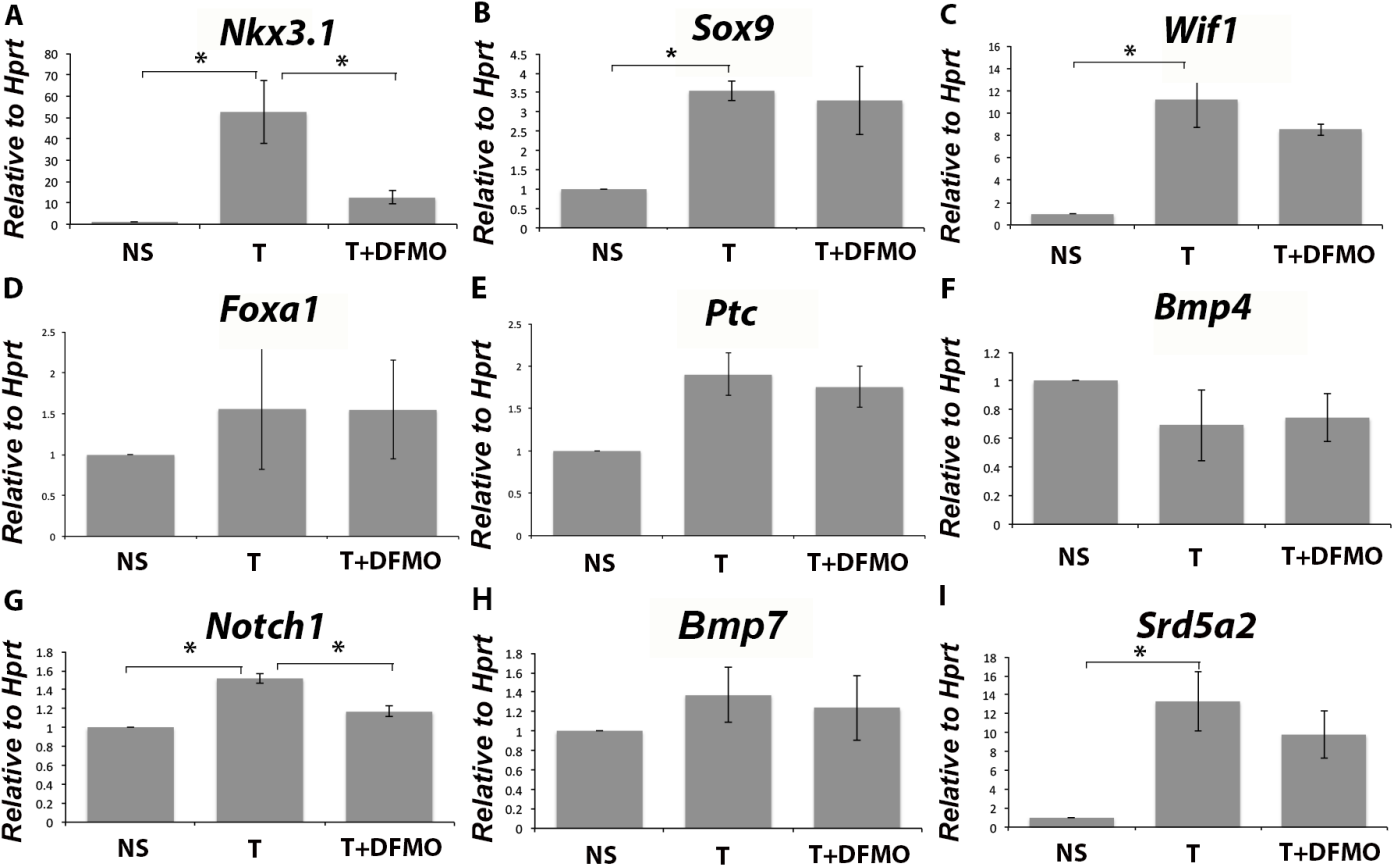
Markers of prostatic budding in cultured UGS tissues. E16 UGS tissues were cultured in the control media, testosterone supplemented media or media with T+DFMO. Testosterone induced the expression of *Nkx3*.*1* (A), *Sox9* (B), *Wif1* (C), *Notch1* (H) and *Srd5a2* (J). It did not induce the expression of *Foxa1* (D), *Shh* (E), *Ptc* (F), *Bmp4* (G) or *Bmp7* (I). Culturing the UGS in T+DFMO significantly decreased expression of *Nkx3*.*1* (A) and *Notch1* (H). * p<0.05.

Prostatic bud initial outgrowth is induced by androgens and coincides with up-regulation of genes. Inhibiting ornithine decarboxylase activity clearly affected prostatic bud formation, so we examined some of the pathways involved in prostatic bud initiation. Testosterone treatment significantly induced the expression of *Nkx3*.*1* (Fig 7A), *Sox9* (Fig 7B), *Wif1* (Fig 7C), *Notch1* (Fig 7G) and *Srd5a2* (Fig 7I), which have previously been shown to be up-regulated in the UGS in response to androgen treatment [6,10,14,48,49]. It did not induce the expression of *Foxa1* (Fig 7D), *Ptc (* Fig 7E), *Bmp4* (Fig 7F) or *Bmp7* (Fig 7H). Inhibiting ornithine decarboxylase with DFMO in the presence of testosterone significantly down-regulated *Nkx3*.*1* (Fig 7A) and *Notch1* (Fig 7G) expression, but did not affect the expression of other factors that were induced by testosterone. The inhibition of certain pathways and not all pathways in prostatic bud formation suggests that polyamines are required for specific pathways, and does not affect global growth and/or differentiation of the urogenital epithelium.

*Nkx3*.*1* is the classical marker of prostatic budding. It is expressed in the urogenital epithelium two days before the onset of prostatic bud outgrowth, in localized areas that will become prostatic buds [11,14]. *Nkx3*.*1* is androgen-regulated, with *Nkx3*.*1* expression decreasing upon castration [14,50]. *Nkx3*.*1* is also required in branching of the salivary gland, as inhibiting *Nkx3*.*1* expression decreases branching in the salivary gland [51,52]. In our study, *Nkx3*.*1* expression was induced by testosterone when the UGS was cultured *in vitro* and significantly decreased after treatment with T+DFMO. There are two possible explanations for this result: either polyamines are required for bud induction, which indirectly affects the regulation of *Nkx3*.*1*, or that the lack of polyamines directly affects *Nkx3*.*1* expression independent of the inhibition of prostatic budding. This is the first report that *Nkx3*.*1* expression changes in response to polyamine depletion.

DFMO treatment also inhibited the testosterone-induced up-regulation of *Notch1* (Fig 7H). *Notch1* is expressed in basal epithelial cells in both the rat and mouse prostate [53]. Ablation of *Notch1* inhibits branching morphogenesis, differentiation and hormone dependent regrowth of the prostate following castration, which suggests that *Notch1* is required for cell proliferation [54]. Inhibiting *Notch1* using a gamma secretase inhibitor in postnatal ventral prostate also decreased tubule formation compared to control [55]. Together, these results suggest that *Notch1* is required for proliferation of the basal epithelial cell population, which likely contribute to the progenitor cell population in the epithelium. In our study, polyamine depletion decreased *Notch1* expression in the urogenital sinus. Similar to *Nkx3*.*1*, polyamines may be required for bud induction, which indirectly affects the regulation of *Notch1*, or that the lack of polyamines directly affects *Notch1* expression independent of the inhibition of prostatic budding.

### Inhibiting polyamines after prostatic bud formation did not rescue *Nkx3*.*1* downregulation

**Fig 8:**
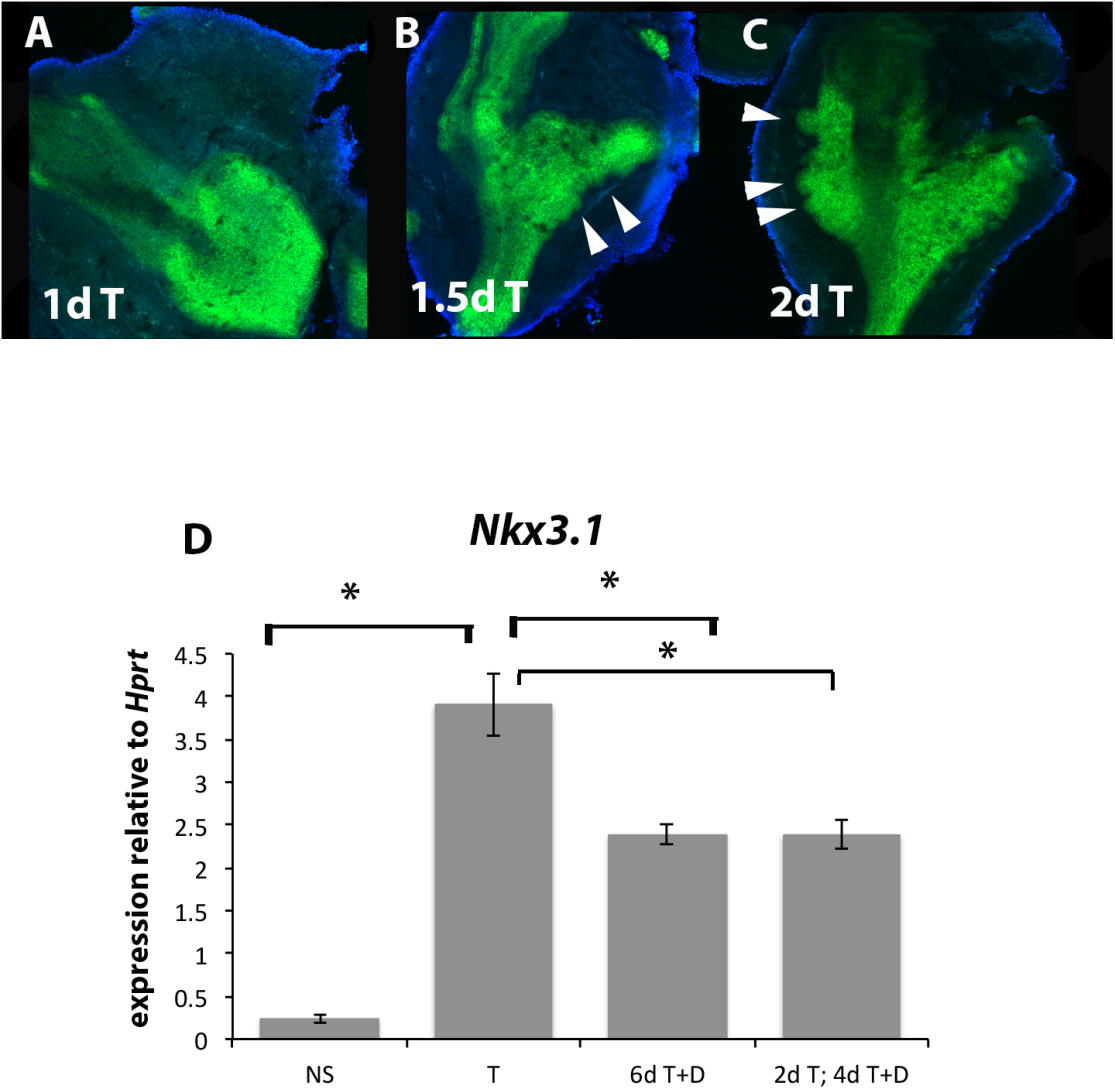
Allowing prostatic buds to grow before polyamine depletion did not rescue *Nkx3*.*1* expression. We determined the time course of prostatic bud formation over 2 days of culture in testosterone, by culturing the UGS in the presence of testosterone for the time indicated, fixed the cultures, and stained them with E-Cadherin to examine prostatic bud formation. After 24 hours or 1 day in culture, prostatic buds were still not apparent (A). After 1.5 days in testosterone, several prostatic buds were observed (B, white arrowheads). The buds were longer and more numerous after 2 days in testosterone (C, white arrowheads). Culturing the UGS in testosterone for two days to allow buds to grow, followed by polyamine depletion did not rescue *Nkx3*.*1* expression (D). *, p<0.05.

We asked whether the reduction in *Nkx3*.*1* is due to the lack of prostatic buds, or whether it is due to a direct effect of polyamine depletion. To do this, we cultured the UGS in testosterone until prostatic buds were first morphologically identifiable, then depleted polyamines using DFMO. Firstly, we determined the period of time required for prostatic buds to become morphologically distinct. Even after six days of culture in no steroid treatment, we did not observe any prostatic buds (Fig 4A). After day 1 of culture in testosterone, we also did not observe prostatic buds (8A). The first morphological buds were distinguished at day 1.5 of culture in testosterone (Fig 8B, white arrowheads) with more buds visible at day 2 of culture in testosterone (Fig 8C, white arrowheads). We then cultured the UGS for two days in testosterone only to allow the prostatic buds to form, followed by polyamine depletion using DFMO for the next four days. Similar to our previous results, *Nkx3*.*1* was significantly up-regulated after 6 days in testosterone compared to NS treatment (Fig 8D, p<0.05) and significantly down-regulated after 6 days in T+DFMO compared to testosterone alone (Fig 8D, p<0.05). However, culturing the UGS in testosterone for two days after prostatic buds have formed, followed by polyamine depletion did not rescue *Nkx3*.*1*, with *Nkx3*.*1* significantly decreased after 2 days of testosterone + 4 days of T+DFMO, compared to testosterone alone (Fig 8D, p<0.05) and no significant difference between 6 days T+DFMO and 2 days T and 4 days T+DFMO. Although inhibiting ornithine decarboxylase after prostatic buds have formed did block *Nkx3*.*1* expression, it did not reduce *Notch1* expression (data not shown). Together, these results suggest that the DFMO-induced reduction of *Nkx3*.*1* was not an indirect effect due to the loss of prostatic buds and that polyamines are directly required to maintain *Nkx3*.*1* expression regardless of the presence or absence of buds.

## Conclusions

Ornithine decarboxylase is the rate-limiting step in polyamine biosynthesis. In the adult mouse prostate, ornithine decarboxylase activity is androgen-dependent, which is evidenced by the decrease in mRNA expression upon castration, and increase in mRNA expression upon androgen replacement [36,45]. We have shown that ornithine decarboxylase is expressed in the male and female urogenital sinus during the period of prostatic bud initiation, suggesting it is not androgen regulated during the initial steps of prostate development. However, inhibiting ornithine decarboxylase activity in the developing prostate inhibited prostatic bud formation and significantly decreased the expression of two key prostate developmental regulatory genes, *Nkx3*.*1* and *Notch1*. Furthermore, inhibiting polyamine biosynthesis after prostatic buds have formed did not rescue *Nkx3*.*1* expression, suggesting that polyamines are directly required for *Nkx3*.*1* expression independent of the budding process. Together our results have shown that ornithine decarboxylase and polyamine synthesis are required for the initial steps of prostate development from the urogenital sinus.

## Supporting Information

S1 Table Primers used to amplify genes of interest in QPCR. (DOCX)

S2 Table Conditions used for antibodies in immunohistochemistry. (DOCX)
