# Supplementary material for: Ornithine Decarboxylase Activity Is Required for Prostatic Budding in the Developing Mouse Prostate
Source: PLoS One. 2015 Oct 1;10(10):e0139522. doi: 10.1371/journal.pone.0139522 (PMC4591331; doi:10.1371/journal.pone.0139522)
Supplement: S1 Table — (DOCX) [file pone.0139522.s001.docx]

S1 Table 1:

QPCR primers for mouse

| Gene name | Forward primer 5’-3’ | Reverse primer 5’-3’ | Amplicon size |
| --- | --- | --- | --- |
| Actb | GAT CAA GAT CAT TGC TCC TCC TG | AGG GTG TAA AAC GCA GCT CA | 183 |
| Arg1 | GAC CTG GCC TTT GTT GAT GT | CTG GTT GTC AGG GGA GTG TT | 254 |
| Amd1 | TTT GAA GGA TGT GCA GTG CT | CCC TAG CAA GCT TCA ACA GG | 170 |
| Bmp4 | ACC CAG CCT GAG TAT CTG GT | GAA TGG CTC CAT TGG TTC CTG | 101 |
| Bmp7 | AGTGCGCCTTCCCTCTGAAC | AGG GCT TGG GTA CTG TGT | 99 |
| Foxa1 | CAT GAG AGC AAC GAC TGG AA | TTG GCG TAG GAC ATG TTG AA | 181 |
| Hprt | GGT GAA AAG GAC CTC TCG AAG | CAA ACG TGA TTC AAA TCC CTG A | 126 |
| Nkx3.1 | CCG AGT CTG ATGCAC ATTTT | CTG TGG CTG CTT GGT GAC | 94 |
| Notch1 | GGT CGC AAC TGT GAG AGT GA | TCG CAG AAG GCT GTG TTG AT | 95 |
| Oaz1 | AGC CGA CGT CTA ACG ACA AG | GTT GCT CCT CTG CGA ACT CT | 183 |
| Odc1 | CAG CAG GCT TCT CTT GGA AC | AGC CAC CAC CAA TAT CAA GC | 184 |
| Paox | AGT CTT CAC ATG TGC TCT GTG GGT | TGG CAA TTG TGG GTT TCC TGT CAC | 131 |
| Ptc | 5'-TGCCGCAGTTCTTTTGAATG-3' | 5'-CTCTGGAGCAGATTTCCAAGG-3' | 117 |
| Sat1 | AAC GAA TGA GGA ACC ACC TCC | CAG GGG TCC AGT GCT CTT TAG | 278 |
| Shh | 5'-AATGCCTTGGCCATCTCTGT-3' | 5'-GCTCGACCCTCATAGTGTAGAGACT | 115 |
| Sms | TCC ACA TGG GAT TTC CTC AG | GCC CCA GCT GTT CTT CAT AG | 133 |
| Sox9 | AGG AAG CTG GCA GAC CAG TA | TGT AAT CGG GGT GGT CTT TC | 156 |
| Srm | TGG TCC AGT GCG AGA TTG ATG | AGT GCT GTC TTC ATG AGC TGG | 232 |
| Wif1 | GAG AAA GCC CTG TGC  ATA CC | ACT GCT CTC TCC CTC GAG TCC | 192 |
