# Supplementary material for: Ornithine Decarboxylase Activity Is Required for Prostatic Budding in the Developing Mouse Prostate
Source: PLoS One. 2015 Oct 1;10(10):e0139522. doi: 10.1371/journal.pone.0139522 (PMC4591331; doi:10.1371/journal.pone.0139522)
Supplement: S2 Table — (DOCX) [file pone.0139522.s002.docx]

S2 Table 2:

Table of immunohistochemistry conditions.

| Antibody | Antigen retrieval | Block | Primary antibody dilution | Secondary antibody |
| --- | --- | --- | --- | --- |
| Ornithine decarboxylase (Sigma O1136) | Diva decloaking antigen retrieval | Rodent Block M (Biocare Medical cat# RBM9616) | 1:100 | Mach-2 mouse secondary antibody (Biocare Medical cat# MALP521G) |
| Ki67 (Abcam 16667) | Vector Unmasking solution 1:100  (Vector labs cat# H-3300) | 2.5% Goat serum in 1XPBS | 1:500 | Goat anti-rabbit |
| WSS cytokeratin (Dako Z0622) | Vector Unmasking solution 1:100  (Vector labs cat# H-3300) | 10% Goat serum in 0.1% BSA/TBS | 1:100 | Goat anti-rabbit |
| SMA (Sigma A5228) | None | MOM block | 1:100-1:200 | Goat anti-mouse |
| p63 (Santa Cruz SC-8341) | Vector Unmasking solution 1:100 (Vector labs cat# H-3300) | MOM block | 1:100 | Goat anti-mouse |
| Vimentin (Abcam) | Vector Unmasking solution | MOM block | 1:50 | Goat anti-rabbit conjugated to Texas Red fluorophore |
| ΔN p63 (N-16, Santa Cruz SC-8609) | Vector Unmasking solution | MOM block | 1:100 | Rabbit anti-goat conjugated to Texas Red fluorophore |
